# Distinct types of glial cells populate the *Drosophila *antenna

**DOI:** 10.1186/1471-213X-5-25

**Published:** 2005-11-11

**Authors:** Anindya Sen, Chetak Shetty, Dhanisha Jhaveri, Veronica Rodrigues

**Affiliations:** 1 Department of Biological Sciences, Tata Institute of Fundamental Research, Homi Bhabha Rd., Mumbai 400005, India; 2 National Centre for Biological Sciences, TIFR, GKVK PO, Bellary Rd., Bangalore 560065, India; 3 Dept. of Physiology and Cellular Biophysics, Columbia University, New York. USA; 4 Queensland Brain Institute, University of Queensland, Brisbane, Australia

## Abstract

**Background:**

The development of nervous systems involves reciprocal interactions between neurons and glia. In the *Drosophila *olfactory system, peripheral glial cells arise from sensory lineages specified by the basic helix-loop-helix transcription factor, Atonal. These glia wrap around the developing olfactory axons early during development and pattern the three distinct fascicles as they exit the antenna. In the moth *Manduca sexta*, an additional set of central glia migrate to the base of the antennal nerve where axons sort to their glomerular targets. In this work, we have investigated whether similar types of cells exist in the *Drosophila *antenna.

**Results:**

We have used different P(Gal4) lines to drive Green Fluorescent Protein (GFP) in distinct populations of cells within the *Drosophila *antenna. Mz317::GFP, a marker for cell body and perineural glia, labels the majority of peripheral glia. An additional ~30 glial cells detected by GH146::GFP do not derive from any of the sensory lineages and appear to migrate into the antenna from the brain. Their appearance in the third antennal segment is regulated by normal function of the Epidermal Growth Factor receptor and small GTPases. We denote these distinct populations of cells as Mz317-glia and GH146-glia respectively. In the adult, processes of GH146-glial cells ensheath the olfactory receptor neurons directly, while those of the Mz317-glia form a peripheral layer. Ablation of GH146-glia does not result in any significant effects on the patterning of the olfactory receptor axons.

**Conclusion:**

We have demonstrated the presence of at least two distinct populations of glial cells within the *Drosophila *antenna. GH146-glial cells originate in the brain and migrate to the antenna along the newly formed olfactory axons. The number of cells populating the third segment of the antenna is regulated by signaling through the Epidermal Growth Factor receptor. These glia share several features of the sorting zone cells described in *Manduca*.

## Background

Odor information in animals is represented as a spatial pattern of activity among glomeruli in the olfactory lobe [1]. This odotopic map is generated by the projection of olfactory receptor neurons (ORNs) each expressing a single odorant receptor (Or) gene to a defined glomerulus(i). How is this wiring pattern achieved? Compelling evidence exists in vertebrates for a role of the Ors themselves in providing cues for connectivity [2,3]. Such a mechanism seems unlikely in insect olfactory development where a carefully orchestrated interaction between ORNs, glial cells and lobe interneurons pattern the structural units underlying odor coding [reviewed in [4]].

The cellular events occurring during development of the *Drosophila *olfactory system have been reviewed recently [5]. Adult ORNs are specified within the antennal disc and project to the brain during early pupation. The neurons travel over the lobe anlage in the outer nerve layer occupying positions specified by interaction of Roundabout receptors with a gradient of the ligand Slit [6]. Axon terminals invade the lobe and project to specific glomeruli where they synapse with local lobe and projection interneurons. An attractive hypothesis is that targeting of ORNs and subsequent synapse formation is regulated by transcripts of the *Down Syndrome Cell Adhesion Molecule *(*Dscam*) gene [5,7].

There are a number of studies that demonstrate interdependence between neurons and glia during development [8,9]. In the *Drosophila *olfactory system, peripheral glial cells have been shown to arise from sensory lineages and play a role in patterning ORNs within distinct fascicles as they exit the antenna [10]. A set of central glia associated with the developing olfactory lobe elaborate projections into the neuropil to ensheath the newly formed protoglomeruli [10]. Ablation of the equivalent cells in the moth *Manduca sexta*, results in a failure in glomerular maturation and stabilization [11,12]. Here the migration of central glia to the glomerular borders is triggered by the earliest arriving ORNs which signal via nitric oxide [13]. In *Drosophila *a group of about 200 neurons, determined by the basic helix-loop-helix (bHLH) transcription factor Atonal (Ato) are the first to enter the lobe and have been proposed to act as pioneers [14]. In their absence the remaining ~1000 neurons fail to make appropriate targets in the lobe. The fate of central glia has not been investigated in these mutants. In *Manduca *an additional group of central glia, termed sorting glia, migrate to the base of the antennal nerve where ingrowing axons sort according to glomerular targets [15]. *In vivo *and culture studies have shown that these glia induce morphological changes in growth cones of the ORNs suggestive of alterations in adhesion and cytoskeletal dynamics [16,17]. This argues for reciprocal signaling between neurons and glia which needs to be investigated further.

In this paper, we demonstrate the presence of a set of cells in *Drosophila*, which share similarities with the sorting zone glia in the moth. The cells which are labeled with GH146::GFP become associated with the antennal nerve when it reaches the brain. The cells migrate into the third segment of the antenna where they ensheath axons of the ORNs as they project from the antenna to the brain. Although evidence that these glia influence the development or the function of the ORNs is lacking, their possible function is discussed with respect to findings in other insects.

## Results

### Cellular markers define two distinct subsets of glial cells within the *Drosophila *antenna

**Figure 1:**
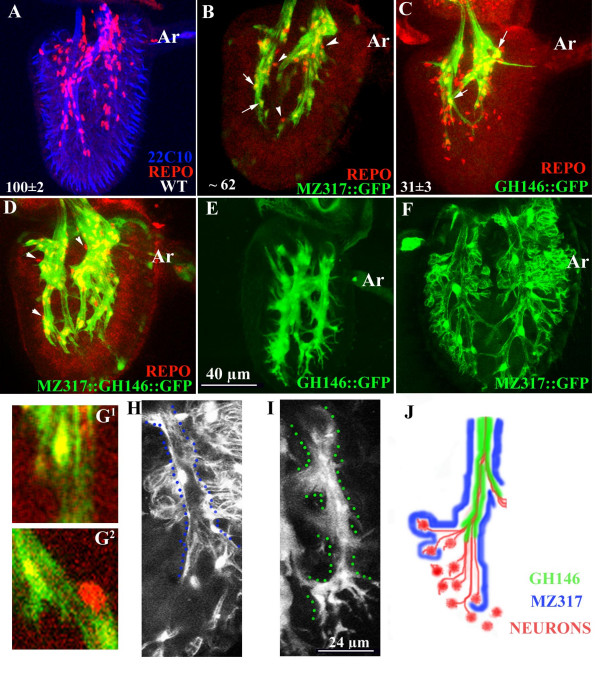
**Two populations of glial cells are present in the third segment of the antenna**. 36 hr pupal antenna stained with the neuron-specific antibody mAb22C10 (blue in A) and anti-Repo (red). (B) Mz317::GFP stained with anti-GFP (green) and anti-Repo. Only a few 1 μm confocal sections have been stacked to show the double labeled cells (small arrows). Repo-positive cells that do not express Mz317-GFP are indicated with arrowheads. (C) GH146::GFP stained with anti-GFP (green) and anti-Repo. Numbers in B (N = 10) and C (N = 3) indicate the GFP expressing Repo-positive cells. (D) 36 hr antenna from GH146::GFP/Mz317-Gal4 pupae stained with anti-GFP (green) and anti-Repo (red). Most of the cells stain with both antibodies except for a few cells indicated with arrowheads. (E) In the late pupa the projections of the GH146-glia are similar to that at 36 hrs APF while those of Mz317 (F) are markedly different. Scale bar = 40 μm for panels A-F; Ar-arista. (G) Magnified region from single confocal sections demonstrating glial cells stained with expressing both Repo and GFP (G^1^) and those expressing Repo alone (G^2^). (H.I) Enlarged view of an axon fascicle in late pupal antennae from Mz317::GFP (H) and GH146::GFP (I) (scale bar = 24 μm). Dotted lines in (I) mark the boundaries of the GH146-glial processes. This region is devoid of Mz317-glial processes. (J) Diagrammatic representation of glial patterning in the adult antenna. GH146-glia (green) tightly wrap around the axon bundles (red) while those of Mz317-glia (blue) are peripheral to this layer.

**Figure 3:**
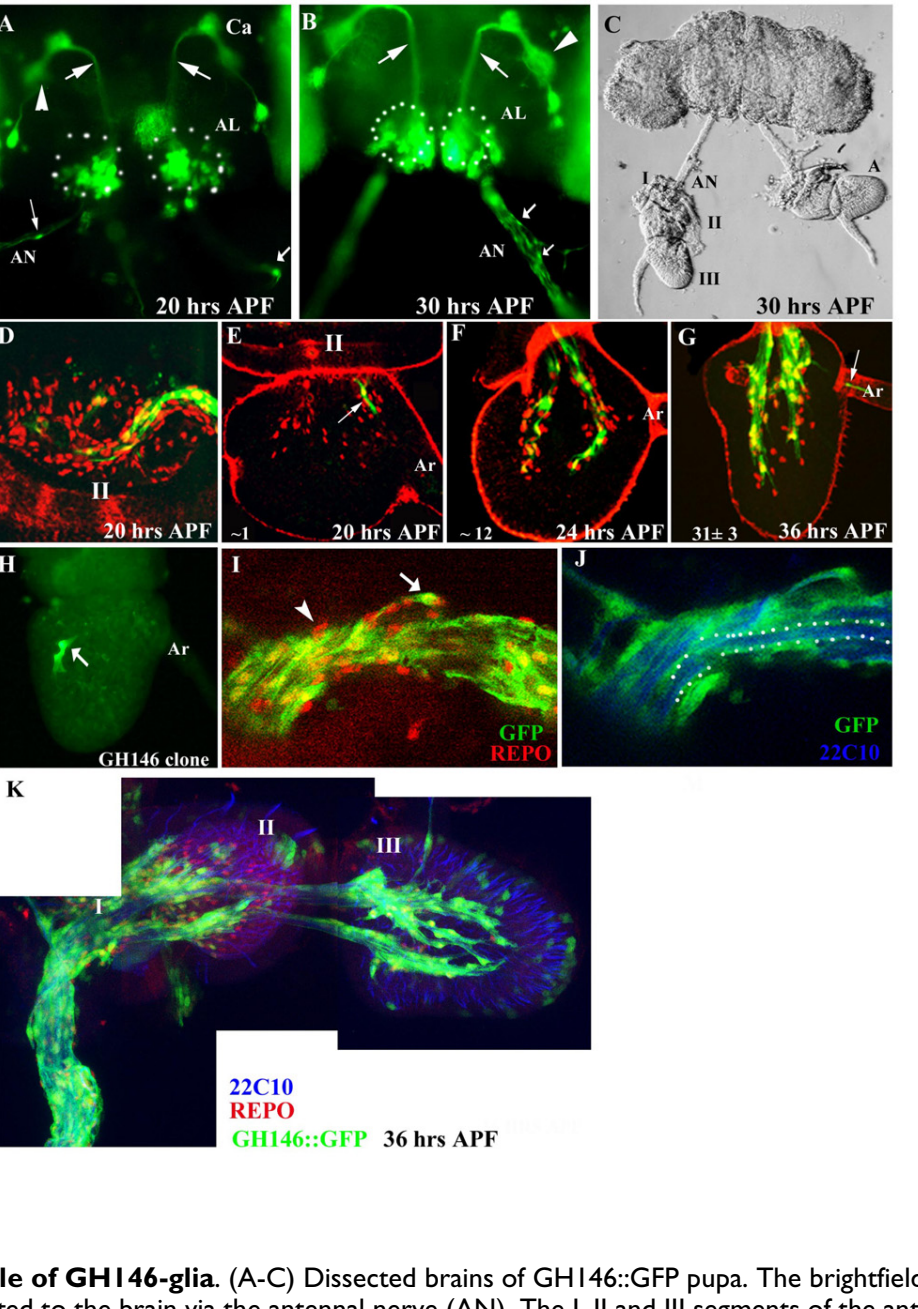
**Developmental profile of GH146-glia**. (A-C) Dissected brains of GH146::GFP pupa. The brightfield image in (C) shows the antenna (A) connected to the brain via the antennal nerve (AN). The I, II and III segments of the antenna are indicated. Fluorescence images of 20 hr and 30 hr preparations are shown in (A) and (B) respectively. GH146::GFP labels interneurons (arrows) that connect the olfactory lobes (AL-dotted circles) to the calyx of the mushroom bodies (Ca) and dorsal horn (arrowhead). A few GFP labeled cells can be seen in the antennal nerve (small arrows) at 20 hrs APF which increase by 30 hrs (small arrows in B). (D-G) Antenna from GH146::GFP pupae stained with anti-Repo (red) and anti-GFP (green). At 20 hrs APF (D,E), a large number of glial cells stained by anti-Repo are present in the second segment (II in D) and only few of these express GFP. Only one or two GH146-glia appears in the third segment (arrow in E). This number increases at 24 hrs APF (F) and 36 hrs APF (G). (H) MARCM clone generated using *ey*-FLP show presence of only two marked GH146-positive cells in the third segment of the antenna (small arrow). (I,J) Antennal nerve of 36 hr APF GH146::GFP pupae stained with anti-GFP (green), anti-Repo (red) and mAb22C10 (blue). The nerve is enlarged in to show the relative positioning of neurons and glia. (I) Only a subset of the nerve associated glia are of the GH146 subtype (arrow). A significant number of Repo-positive cells do not express GFP (arrowhead). (J) Processes of GH146-glia segregate axons into distinct bundles (dotted lines). (K) 36 hr APF antenna and attached antennal nerve to show the processes of the GH146-glia (green) with respect to the neurons stained with mAb22C10 (blue) and glial cell bodies marked with anti-Repo (red). The positions of the I II and III segment of the antenna are marked.

We stained pupal antenna with antibodies against the transcription factor Reversed Polarity (Repo) which serves as a marker for differentiated glial cells. Repo-positive cells are first detected at about 16 hours (hrs) after pupa formation (APF) when they closely associate with developing sensory axons [10,14]. At 36 hrs APF sensory neurons have differentiated (blue in Fig. 1A) and the third segment is invested with 100 ± 2 glial cells (red in Fig. 1A). The use of P(Gal4) lines to drive GFP in subsets of cells allows us to distinguish two classes of antennal glia. We constructed stocks carrying recombinant chromosomes bearing the UAS-GFP transgene with either *Mz317*-Gal4 (Mz317::GFP) or *GH146*-Gal4 (GH146::GFP). Mz317::GFP is known to mark peripheral, perineural and cell body glia [18] and labels about 70% of glial cells which we denote Mz317-glia (Fig. 1B). GH146::GFP, is a widely used marker for projection interneurons which connect the olfactory lobes to higher centres in the calyx of the mushroom bodies and the lateral horn [[19]; see Fig. 3A,B]. This marker labels about 30% of Repo-positive cells in the third antennal segment which we refer to as GH146-glia (Fig. 1C). In order to demonstrate that these reporters mark non-overlapping glial populations, we stained antennae from pupae carrying both Mz317::GFP and GH146::GFP transgenes with anti-GFP and anti-Repo (Fig. 1D). The co-localization of Repo positive cells with GFP expressed was evaluated carefully in 1 μm confocal sections through the entire antenna (Fig. 1G). All the Repo-positive cells barring a small set of 12 ± 3 (n = 6) (arrowheads in Fig. 1D) were labeled by the GFP reporters. These data together with that presented in Figures 1B and 1C suggest that antennal glial cells are either of the Mz317 or the GH146 type. The cells unlabeled by either reporter could represent yet another class of cells or could reflect lower levels of expression of GFP of one of the Gal4 drivers. This needs to be examined further.

At 36 hr APF, all the glial processes tightly ensheath fascicles of sensory axons as they exit the antenna (green in Fig. 1D). The GH146-glia remain in the same position up to adulthood (Fig. 1E), while projections of Mz317-glia appear to 'loosen' from the fascicles to ensheath the cell bodies of the peripheral sense organs (Fig. 1F). A careful comparison of GFP labeled processes in Mz317::GFP (Fig. 1H) and GH146::GFP (Fig. 1I) suggest that the latter ensheath the neurons directly while Mz317-glia form an outer layer (schematized in Fig. 1J).

### What is the origin of the antennal glia?

**Figure 2:**
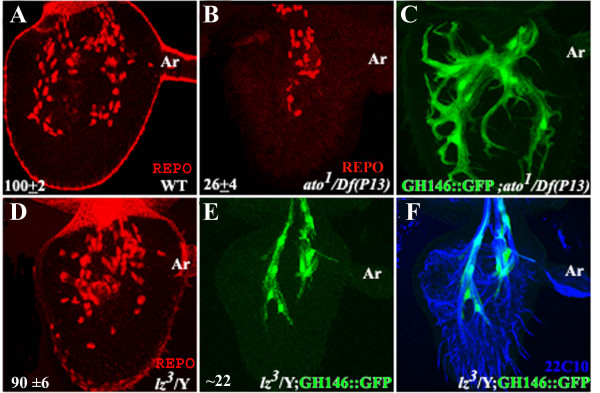
**GH146-glia does not originate from the sensory lineages**. (A) Third antennal segment from 36 hr APF wildtype pupae stained with anti-Repo showing the position of glial cells. (B) 36 hr APF *ato*^*1*^/*Df(3R)p*^*13 *^antenna shows a significant reduction of glial cells, compared to the wildtype. (C) GH146::GFP was crossed into *ato*^*1*^/*Df(3R)p*^*13 *^to visualize GH146-glia in the late pupa. (D) 36 hrs APF *lz*^3 ^antenna stained with anti-Repo. (E). *lz*^3^; GH146::GFP antenna stained with anti-GFP. (F) Antenna in (E) was also stained with mAb22C10. Sensory neurons are markedly reduced in number. Ar-arista. Numbers in panels A,B and D represent the mean and standard deviation of glial cell number in at least 8 antenna. For E only 5 antennae were counted.

There are three morphologically distinct types of sense organs on the antennal surface- the coeloconica, trichoidea and basiconica [20]. Progenitors of the approximately 70 coleoconic sensilla which are specified by Ato, each give rise to a glial cell [10,20]. A second b-HLH protein Amos, in combination with the Runt family transcription factor Lozenge (Lz), specifies the trichoidea and basiconica [21,22]. Amos-dependent lineages are also gliogenic but nascent glial cells undergo programmed cell death [23]. We stained 36 hr APF antennae from animals null for *ato *(*ato*^1^/Df(3R)*p*^13^) with anti-Repo (Fig. 2B). Only 30% of the ~100 cells were formed (Fig. 2A,B). GH146::GFP was crossed into this genetic background to demonstrate that the extant cells were all of the GH146-glial subtype (not shown). In late pupae, projections of GH146-glia are more extensive than in the wildtype (compare Fig. 2C with 1E). We believe that the aberrant morphology of projections could be explained by a lack of Mz317-glia which could restrict the extension of GH146-glial projections in normal animals.

We next examined whether the GH146-glia could be a subset of Amos-dependent glial cells which escape apoptosis. The majority of Amos-dependent sensilla fail to form in strong *lz *alleles. We stained 36 hr antenna from *lz*^3 ^pupae with anti-Repo and found that the number of cells observed was not significantly different from that of wildtype controls (p < 0.05; Fig. 2D). In animals where GH146::GFP was crossed into this background these glia were observed (Fig. 2E). The somewhat reduced number of cells as compared to the wildtype can be explained by the strong decrease in ORN number [21], assuming GH146-glial cells require axons to navigate into the antenna (discussed below).

The observation that GH146-glia appears in both *ato *and *lz *mutants leads us to suggest that the cells do not arise from sensory lineages but probably 'home' into the antenna from elsewhere.

### The time course of appearance of GH146-glia suggests migration into the third antennal segment

Since the GH146-glial cells do not have a peripheral origin, we decided to examine the possibility that they migrate into the antenna from the Central Nervous System (CNS). Pupal dissections exposing both antenna and brain (Fig. 3C) were stained with anti-GFP and anti-Repo to observe the appearance of glial cells at different pupal ages (Fig. 3A,B). The first cells are seen on the antennal nerve at 20 hrs APF (small arrows in Fig. 3A), a time corresponding to the entry of olfactory neurons into the brain [10]. One or two GFP positive cells are also detected within the third segment of the antenna at this time (arrow in Fig. 1E). The number of cells associated with the nerve increases steadily with pupal age (not shown) up to about 30 hrs APF (compare small arrows in Fig. 3B with 3A). GH146::GFP positive cells also increase within the third antennal segment to reach a maximum of ~30 at 36 hrs APF (Fig. 3E–G). The GFP labeled cells are only a small fraction of total glial cells detected by anti-Repo staining within the second (Fig. 3D) and third antennal segment (Fig. 3E–G). A few cells can also be detected within the arista (arrow in Fig. 3G).

While these observations support the idea that GH146-glia migrate into the third segment along the olfactory neurons, it is possible that cells already located within the antenna turn on the GH146 enhancer in a dynamic fashion. We exploited the MARCM method [24] to further investigate the origin of GH146-glia. Flipase driven by the *eyeless *(*ey*) promoter (ey-FLP) generates a high frequency of large clones covering at least half of the antennal tissue [25,26]. In a control experiment using a marker for ORNs (Or83b::GFP), we obtained >90% clonal frequency (45/49; Pinky Kain personal communication). We reasoned that if, like the ORNs, the GH146-glial cells originate within the antenna, we would obtain a similar frequency of clones. We however obtained only a 15% (3/20) clonal frequency and these were very small covering only two or three cells in each case (arrow in Fig. 3H). These animals had large clones of marked cells within the brain. A possible interpretation is that FLP-mediated recombination generated GH146::GFP cells in the brain and some of these cells migrated peripherally into the antenna.

Data presented in this section, leads us to suggest that the GH146-glial cells arise in the brain and migrate along the nascent olfactory axons to line the fascicles within the third antennal segment. These cells are however, only a small subset of the nerve associated cells and both GFP (arrow in Fig. 3I) and non-GFP (arrowhead in Fig. 3I) Repo-positive cells are detected. Staining of the mature antennal nerve with mAb22C10 and anti-GFP revealed that projections of GH146-glia segregate groups of ORNs as they project to the brain (Fig. 3J,K).

### What are the mechanisms that control GH146-glial number in the antenna?

**Figure 4:**
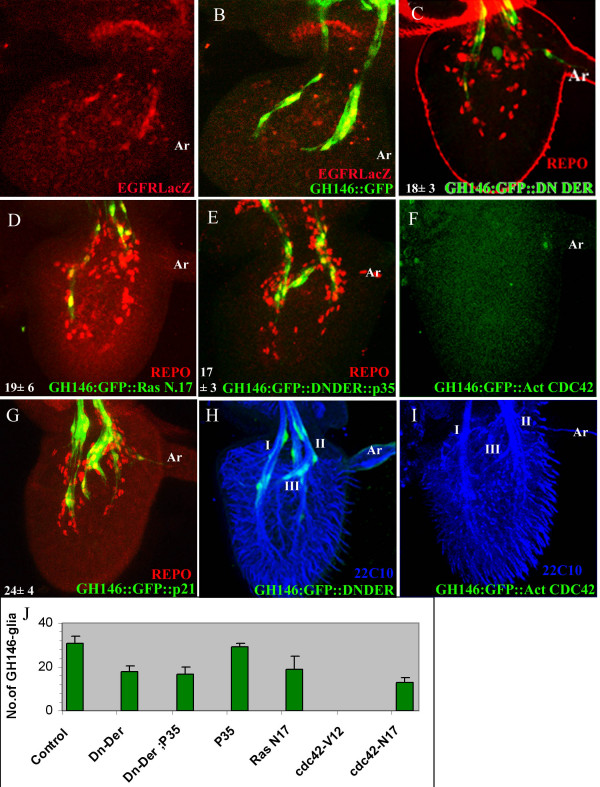
**Factors regulating the numbers of GH146-glia within the third segment of the antenna**. (A,B) GH146::GFP /DER-lacZ stained with anti-β-galactosidase (red in A,B) and anti-GFP (green in B). Merged image in (B) shows that GH146-glia express the DER. (C-G). 36 hr APF antenna stained with anti-GFP (green) and anti-Repo (red). Cell numbers shown in each panel are from at least 10 antenna. (C) GH146::GFP/UAS-DN-DER. (D) UASRas^N17^/+; GH146::GFP/+ (E) GH146::GFP/UAS-DN-DER; UAS-P35/+ (F). GH146::GFP/UAS-Cdc42^v12 ^(G) UAS-p21/+; GH146::GFP/+. (H,I) 36 hour APF antennae of GH146::GFP/UAS-DN-DER (H) and GH146::GFP/ UAS-Cdc42^v12 ^stained with mAb22C10 (blue). ORNs exit the third segment of the antenna in three fascicles denoted I, II and III. The slightly distorted fasciculation pattern seen in (I) is within normal variation. The arista (Ar) is to the right in each figure. (J) Numbers of GH146-glia in antenna expressing different transgenes. Cells in 10 antennae were counted in each case and the mean and standard deviation is represented.

Several studies have demonstrated that glial cell number is regulated by a combination of cell migration, proliferation and programmed cell death. Aigouy and colleagues [27] have followed glia cells as they migrate along the axons in the wing blade and observed dramatic cytoskeleton changes and proliferation during migration. The signals for migration as well as glial cell survival arise from the epidermal growth factor receptor (DER) pathway [28] which impinges upon the small GTPases Ras, Rho and Rac [29-31]. We crossed a DER-lacZ transgene into the GH146::GFP strain and stained 36hr APF antenna with antibodies against β-galactosidase and GFP (Fig. 4A,B). The presence of the DER reporter in GH146-glial cells demonstrates that these cells expressed the receptor at some time in their developmental history. Ectopic expression of a dominant negative form of DER (DN-DER) in these glia (GH146::GFP/UAS-DNDER resulted in a significant reduction in the numbers of GH146-glial cells (Fig. 4C; P < 0.01). Since DER signals through the Ras/MAPK kinase pathway, we confirmed the requirement for this pathway by ectopically expressing a dominant negative form of Ras- Ras^N17 ^(Fig. 4D). The phenotype observed would be expected if abrogation of DER signaling affects survival, proliferation or migration of GH146-glial cells. Programmed cell death can be inhibited by expression of the Baculovirus Inhibitor of Apoptosis -P35. When P35 was co-expressed in GH146-glia together with DN-DER there was no rescue of glial cells as compared to expression of DN-DER alone (Fig. 4E; P < 0.0001). We ascertained that expression of P35 alone did not affect GH146-glial cells. The cell number was not distinguishable from that in the wildtype (P < 0.001; Fig. 4J). These data together indicate that failure of DER signaling in GH146-glial cells does not affect cell survival, thus indicating defects in proliferation and/or cell migration.

Cell migration is regulated by signaling to the cytoskeleton by small GTPases, Rac, RhoA and Cdc42 [31]. Ectopic expression of dominant negative and constitutively active forms of Cdc42 leads to a dramatic reduction in GH146-glia (Fig. 4J). The effect was particularly striking with Cdc42^v12^; no GH146-glia was detected within the third segment of the antenna (Fig. 4F). These data lend some support to the hypothesis that GH146-glia migrate into the third antennal segment probably from the CNS. The morphology and arrangement of GH146-glia suggests that these cells undergo division during migration along the antennal nerve. We blocked cell division by ectopic expression of the human cyclin-dependent kinase inhibitor p21CIP1/WAF1 in the GH146 lineage. We noticed a significant reduction (Fig. 4G; P < 0.01) in the number of these cells within the antenna. These data do not allow us to distinguish whether the cells undergo proliferation during migration or upon reaching their final destination.

### What is the consequence of a lack of GH146-glia on the ORNs?

ORNs exit the antenna towards the brain in three well defined fascicles [[10], Fig. 4H). Previous work had shown that constitutive activation of small GTPases in Mz317-glia compromises the patterning of sensory axons [10]. In order to test whether GH146-glial cells play a similar role, we stained 36 hr antennae from animals in which these cells were either reduced (Fig. 4H) or absent (Fig. 4I), with the neuron-specific antibody mAb22C10. The pattern of ORNs was not significantly altered in either case; the small irregularities seen in Figure 4I are within normal variation.

**Figure 5:**
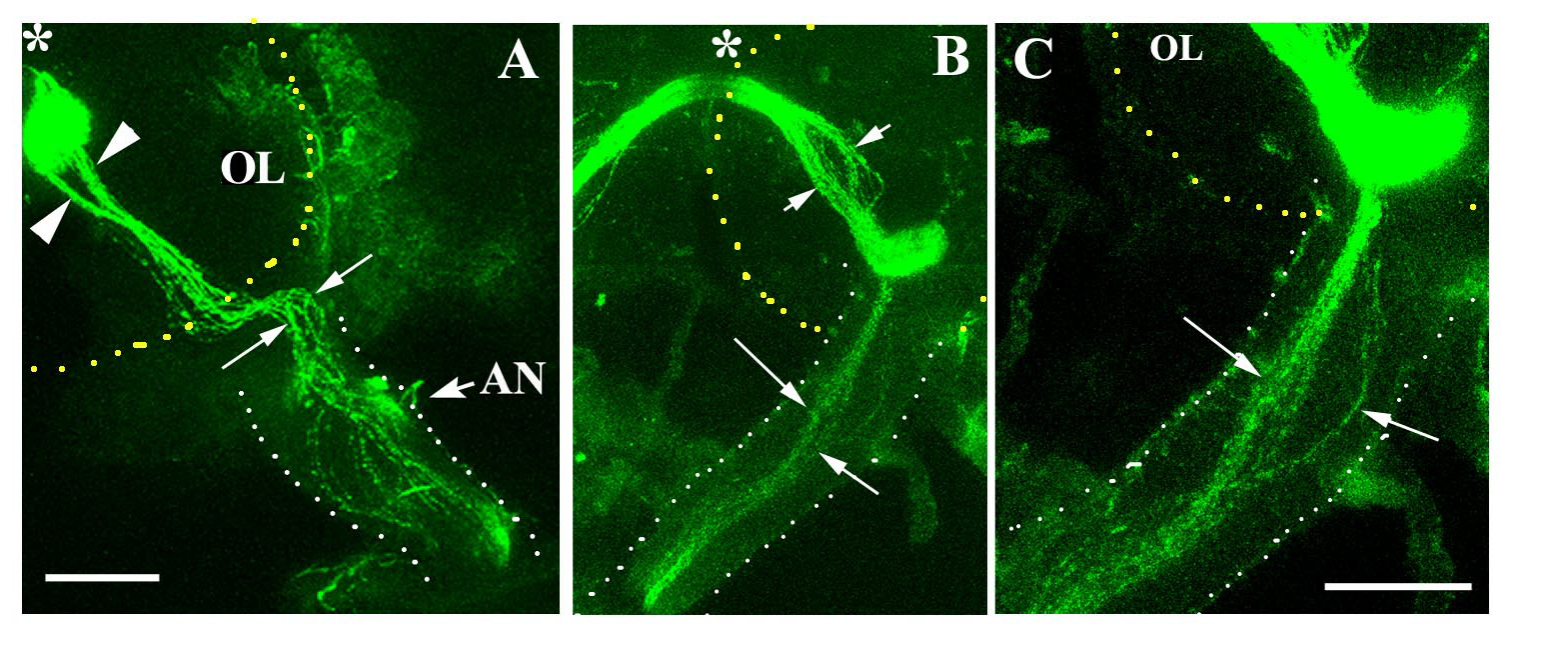
**Positioning of functionally similar olfactory neurons within the antennal nerve**. Dissected brains of Or22a-Gal4;UAS-N-SybGFP (A) and Or47b-Gal4;UAS- N-SybGFP (B,C) were stained with antibodies against GFP. The antennal nerves (AN) are marked with white dots and the boundaries of the olfactory lobe (OL) by yellow dotted lines. Or22a-neurons travel in different regions of the antennal nerve (thin arrows) to enter the DM2 glomerulus in two bundles (arrowheads). Similarly Or47b axons traverse the nerve to reach VA1 glomerulus and also cross the midline (marked in *) to the contralateral lobe. The image in C is magnified (scale bar = 30 μm) and shows that neurons expressing the same Or gene do not group together within the lobe. Scale bar = 40 μm in A, B.

In *Manduca*, glial cells that migrate to the base of the antennal nerve act to sort ORNs to different glomeruli [15]. GH146-glial cells in *Drosophila *move from the CNS to third segment of the antenna. If this position marks a 'sorting zone', one would expect sensory axons to be patterned within the nerve according to their glomerular targets. Processes of GH146-glia have been shown to segregate sensory axons into groups within the antennal nerve (Fig. 3J). ORNs expressing a given Or project to the same glomerulus in the olfactory lobe [32], although these axons use different fascicles within the third antennal segment to enter the nerve [33]. Do functionally similar axons group together within the nerve? We examined 1 μm confocal sections through the antennal nerve of Or22a::GFP and Or47b::GFP animals stained with antibodies against GFP. Axons of ORNs expressing a given Or are positioned differently within the antennal nerve as they travel the brain (small arrows in Fig. 5A–C). Staining with mAb22C10 showed the presence of several other neurons intermingled between those of Or22a and Or47b (not shown). In the case of Or22a, the axons project to the DM2 glomerulus in two distinct fascicles (arrowheads in Fig. 5A).

Hence while bundles of axons are segregated within the antennal nerve by projections of GH146-glial cells (Fig. 3I–K), this fasciculation does not appear to be based on Or identity. The functional significance of this axonal grouping needs to be investigated further.

## Discussion

**Figure 6:**
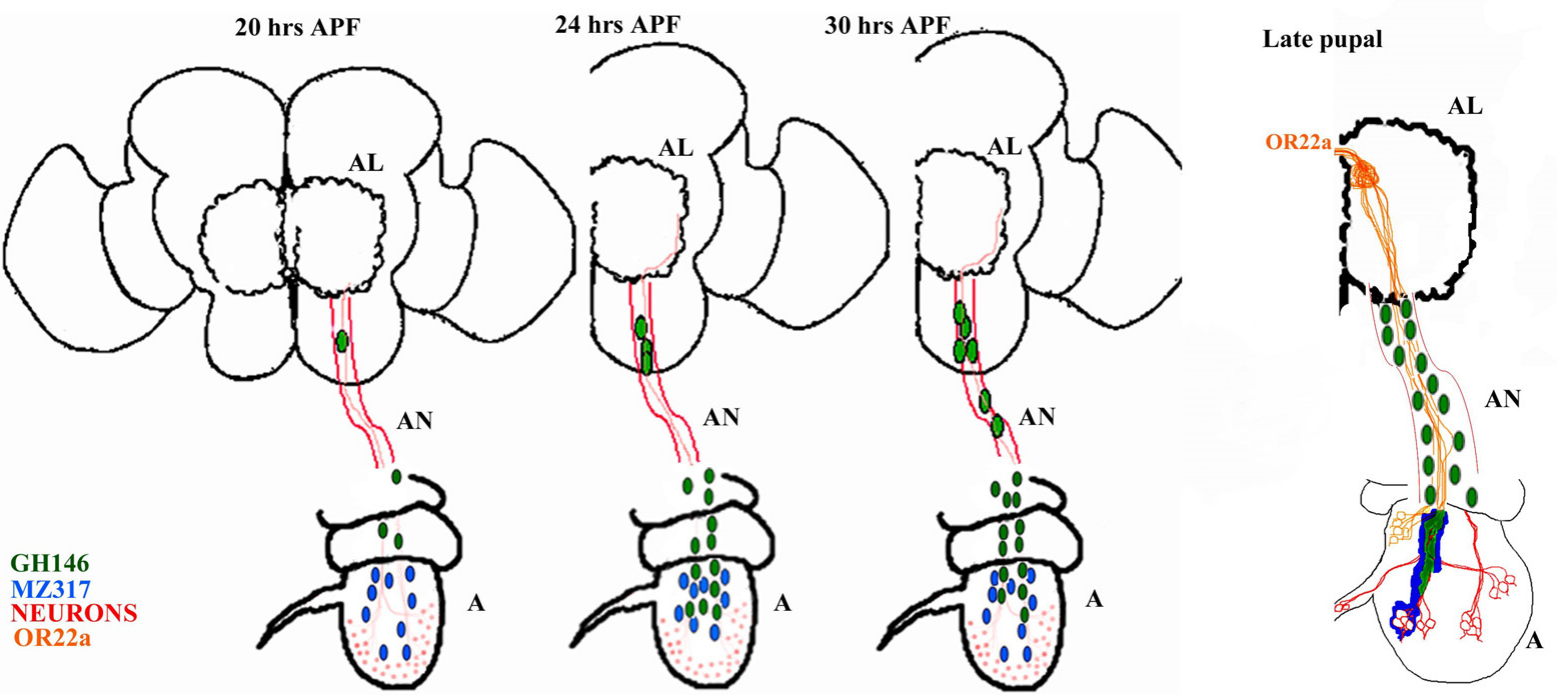
**Model for the migration of the GH146-glia into the antenna**. Olfactory axons arrive at the brain at 20 hrs APF. At this time the Mz317-glia (blue) are already present within the third segment and are associated with developing axons. GH146-glial cells (green) appear on the antennal nerve (AN) when it arrives in the brain. By 24 hrs additional glia are observed on the nerve; the morphology of the cells suggest that they undergo mitosis as they travel along the nerve. By 30 hrs APF, large numbers of cells are present on the nerve and these some of these enter the third segment of the antenna. The cell bodies of Mz317-glial cells appear to move towards the cell bodies of the ORNs at this time. In late pupae, GH146-glia remain closely associated with the neuronal fascicles while Mz317-glial processes appear to move away and wrap around the cells bodies of the sense organs. ORNs expressing Or22a do not appear to fasciculate together in the *Drosophila *antennal nerve.

Studies in *Manduca*, have described three types of glia associated with the developing olfactory neuropil and nerve: i) peripheral glia that arises from the antenna and ensheath olfactory axons [16]; ii) lobe neuropil glia that surround and stabilize olfactory glomeruli [11,12]; iii) sorting zone glia which are central in origin and migrate to the base of the antennal nerve where they serve to segregate axons that target independent glomeruli [15,17]. In *Drosophila*, previous work has described the direct equivalents of peripheral antennal and the neuropil glia [10]. In this paper, we identify an addition set of glia marked by GH146::GFP that share common features with sorting zone glia. Our model, depicted in Figure 6, suggests that these glia arise in the brain and migrate along the ORNs which have newly connected to the brain. Cells proliferate as they migrate along the nerve and enter the third segment of the antenna. Coincident with GH146-glia arrival at the periphery, the Ato-derived glia marked by Mz317::GFP which are already present on the olfactory axons move to wrap around the cell bodies of the sensory organs. The GH146-glial processes tightly ensheath the olfactory fascicles and form a layer below that of the Mz317-glial projections. In all 36 hrs APF preparations examined, we detected ~30 GH146-glia suggesting there must exist mechanisms to regulate the number of cells terminating in the antenna.

### What is the function of GH146-glia during olfactory development?

Although these cells share many of the properties of sorting zone glia described in the moth, we were unable to implicate them in any guidance function. In *Manduca*, Fasciclin II (FasII), the insect ortholog of N-CAM, is expressed in a subset of ORNs which are scattered throughout the antennal nerve. Upon reaching the glial rich sorting zone, these projections segregate from the non-expressing axons and terminate in distinct glomeruli [34]. In *Drosophila*, we were unable to detect the presence of FasII protein on the ORNs during axon projection using the available antibodies (unpublished observations). When the GH146-glia were completely absent within the third antennal segment, we could not detect significant abnormalities in ORN fasciculation within the antenna. The processes of the GH146-glia extend into the antennal nerve to ensheath axon bundles. This segregation is not based on Or gene type and its functional significance is obscure. A similar migration of glial cells from the centre into the periphery has been described during development of the *Drosophila *compound eye [35].

### What are the factors that induce migration of glia from the brain towards the periphery?

Cell migration in insect glia is well known to be mediated by signaling through the Fibroblast Growth Factor Receptors (FGFRs) [36]. Our preliminary observations (unpublished) appear to rule out a requirement for FGFR signaling during GH146-glial migration. The downstream effector of FGF (Dof), is not expressed in these cells at any time during their development. Further, expression of dominant negative forms of *Drosophila *FGF receptors do not affect glial cell number in the antenna (our unpublished data).

We suggest that GH146-glial migration is mediated by EGF signaling [37]. The ligand for DER, Vein has been shown to be known to be expressed in the antennal epidermis at a time consistent with the arrival of cells from the centre. The mechanisms involved in triggering this homing of cells into the antenna require extensive investigation.

## Conclusion

We have identified a new population of glial cells in the antenna of *Drosophila*. The cells originate centrally and use the newly targeted olfactory axons to migrate to the third segment of the antenna. The signal for migration appears to involve EGF signaling. The data in this paper support the idea that in *Drosophila *there is no discrete sorting region equivalent either to the sorting zone in *Manduca *or to the inner nerve layer in the vertebrate olfactory bulb.

## Methods

### Fly strains

*Mz137*-Gal4 was kindly provided by Kei Ito and *GH146*-Gal4 by Reinhard Stocker. These lines were used to drive UAS-GFP (1010T2) thus labeling peripheral glia. The *ato *strains *ato*^1^/TM3 and Df(3R)*p*^13^/TM3 were obtained from Andrew Jarman [14]. The Epidermal growth factor receptor (EGFR)-lacZ strain, UAS-DN DER, UAS-Ras^N17^, UAS-Cdc42^v12^, UAS-Cdc42^N17^, UAS-P35, UAS-p21, *yw*;P [*ry*^+^t7.2-neoFRT}19A, Tub-Gal80 P[*ry*^+^t7.2-neoFRT}19A and *ey*-FLP were obtained from the Bloomington Stock Centre at Indiana, USA. *Or22a*-Gal4, *Or47b*-Gal4 and Or83b-Gal4 lines were kindly provided by Leslie Vosshall [32].

All flies were reared at 25°C on standard cornmeal media containing yeast. For staging, white prepupae (0 h after puparium formation, APF) were collected and allowed to develop on moist filter paper. This stage lasts for an hour; hence the error in staging is 30 minutes. Ages of pupae grown at other temperatures were normalized with respect to growth at 25°C. Wild type pupae take about 100 hrs to eclose when grown at 25°C in our laboratory.

### Immunohistochemistry

Pupal tissues were dissected in phosphate-buffered saline (PBS) and treated as described in [5]. The primary antibodies used were mAb22C10 (1:50, DSHB donated by Seymour Benzer), Rabbit-anti-Repo (1:1000, Susinder Sundaram), Rat-anti-Repo (1:500, Susinder Sundaram), Rabbit anti-GFP (1:10,000, Molecular Probes). Secondary antibodies used were: Alexa 488 goat anti-rabbit, Alexa 568 goat anti-mouse, Alexa 568 goat anti-rat (Molecular Probes) at 1:400; Cy5 conjugated goat anti-mouse (Amersham); at 1:300. The fluorescently labeled preparations were mounted in Vectashield (Vector Labs) and viewed on a BioRad MRC1024 or Radiance 2000 confocal microscope. Two-dimensional projections were generated by stacking appropriate sections for each channel using Confocal Assistant software (distributed by Bio-Rad). Image processing, including pseudo-coloring and labeling, were done using Adobe Photoshop 7.0.

### Imaging of late pupal antennae

Antibody staining of the late pupal stages cannot be carried out because of the hardening of the cuticle. To visualize GFP fluorescence, antennae were removed, mounted in 70% glycerol and scanned on a Radiance 2000 confocal microscope immediately.

### Generation of clones for lineage analysis

Clones were generated using the mosaic analysis with repressible cell marker (MARCM) method described in [25]. Pupae of genotype FRT19A/*Tub*-Gal80 FRT19A; *GH146*-Gal4 UAS-GFP/+; *ey*-FLP/+ and FRT19A/*Tub*-Gal80 FRT19A; *Or83b*-Gal4 UAS-GFP/+; *ey*-FLP/+ were dissected and antennae and brains were stained with antibodies against GFP. Preparations were examined by confocal microscopy for GFP positive cells.

## Authors' contributions

AKS carried out the characterization of the Mz317 and GH146 glia in the antenna and studied the effect of DER and small GTpases signaling. CS established the migration of glia by following expression in dissected preparations and by clonal analysis. DJ carried out the initial observations on the GH146. VR conceived and designed the study, participated in drafting the manuscript. All authors have read and approved the final manuscript.
